# Supplementary material for: Are carbon emissions trading and green financial instruments synergistic? -Comprehensive quantitative research based on content analysis
Source: PLoS One. 2024 Mar 7;19(3):e0298601. doi: 10.1371/journal.pone.0298601 (PMC10919601; doi:10.1371/journal.pone.0298601)
Supplement: S1 Data — (ZIP) [file pone.0298601.s001.zip › Data/地级市Z_F工作报告.zip!/║■▒▒/╦μ╓▌╩╨/└┤╫╘╣1⁄2╓┌║┼í╛┬φ┐╦╩2╛▌═°í┐_2019.docx]

各位代表：

　　现在，我代表市人民政府向大会报告工作，请予审议，并请各位政协委员和列席人员提出意见。

一、2018年工作回顾

　　刚刚过去的一年，面对经济稳中有变和风险挑战明显增多的严峻形势，我们坚持以习近平新时代中国特色社会主义思想为指引，深入贯彻习近平总书记视察湖北重要讲话精神，紧紧围绕省委省政府决策部署，紧扣高质量发展根本要求，紧密团结依靠全市广大干部群众，保持定力、感恩奋进，精准发力、稳中求进，经济社会持续健康发展。全市地区生产总值突破千亿元大关，实现历史性跨越。随州被纳入国家淮河、汉江生态经济带发展规划，构筑鄂北生态屏障纳入省级战略，全省“一芯驱动、两带支撑、三区协同”战略布局提升我市新定位，建设品质随州明晰发展新路径。

　　（一）实体经济稳中有进。出台支持民营经济发展20条、工业经济稳增长快转型高质量发展等系列政策措施，开展“大调研、大走访、大落实”活动，设立1亿元产业基金，助推实体经济行稳致远。主要经济指标平稳增长，地区生产总值增长7.8%，规模工业增加值增长8.1%，固定资产投资增长11.2%，社会消费品零售总额增长11%，实际利用内联资金、利用外资分别增长10%和8.1%。曾都区被授予全省县域经济工作成绩突出单位。工业经济企稳向好。制定专汽、农产品加工、生态文化旅游产业培植方案，开展重点企业“五个一”服务，推行周六现场办公制。实现工业总产值1378亿元，工业用电量、货运量分别增长8.2%和10.5%。专汽产业逆势上扬，产值增长16.5%，产量突破13万辆，新增资质企业4家；中国专汽之都通过复审，我市被授予全国机械工业引领高质量创新发展产业集聚区，齐星家庭化重卡、恒天新能源汽车、江南高端消防车、三铃电力维护车、程力军工系列等新产品竞相推出，不断改写随州专汽发展史。农产品加工业持续壮大，出口额居全省首位，获评中国特色农产品优势区。新能源产业快速发展，并网装机容量突破200万千瓦，累计发电量占全省近30%。农业基础更趋稳固。粮食生产保持稳定，建成一批“集中制袋、分散出菇”生产基地，规模化种植突破3000万袋，香菇酱等系列产品成功研发，“随州香菇”入列全省农产品区域公共品牌二十强。新发展农民合作社721家、家庭农场154家，新增耕地流转7.2万亩，虾稻共作面积达到10.65万亩。随县获批创建全国农村一二三产业融合发展先导区，广水市获评全国农村创业创新典型县范例，市供销社成为全国供销合作社系统先进集体。文化旅游稳步发展。炎帝故里文化旅游区创5A通过省级验收，被授予全国研学实践教育基地。千年银杏谷和编钟乐舞分别获评2018《魅力中国城》年度魅力生态景区、魅力旅游演艺节目。戊戌年世界华人炎帝故里寻根节、首届“中国农民丰收节”湖北主会场活动成功举办，乡村赏花游品牌叫响全省。大洪山新区重点工程启动建设，洛阳镇胡家河村、淮河镇龙泉村、武胜关镇桃源村等民宿示范点各具特色，工业游、度假游和田园综合体等旅游新业态加快成长。全年接待游客2500万人次，旅游综合收入155亿元，分别增长12%和10%。

　　（二）三大攻坚战扎实有力。防范化解重大风险，政府债务、金融风险有效防控，开展扫黑除恶专项斗争，侦破案件135件，抓获犯罪嫌疑人263人，实现“六个没有发生”。特色种养、光伏、电商、旅游等产业扶贫成效显著，教育、健康、社保兜底等政策全面落地，易地扶贫搬迁、安全饮水工程提前完成，全市3.1万人脱贫、24个贫困村出列。践行绿水青山就是金山银山理念，在全省率先成立12个环保专业委员会，开展生态环保九大攻坚行动，4个国考断面和3个县级以上集中式饮用水源地水质全部达标，PM2.5均值持续下降，优良天数比例79.9%，空气质量是近年来的最好时期。中央环保督察“回头看”交办问题基本办结，省环保督察交办问题整改销号75%。

　　（三）质量效益明显提升。收入结构趋优。按新政策口径完成地方公共财政预算收入47.38亿元，增长9.4%；税收占比76.4%，收入质量居全省前列。工业企业主营业务收入增长8%。城乡居民人均可支配收入增速与经济发展同步。转型升级加快。实施“百企百亿”技改工程，17家企业跻身全省支柱产业细分领域隐形冠军，新增4家省智能制造试点示范企业。战略性新兴产业增加值增长8.2%，电商交易额增长20%。创新驱动发力。各类创新平台发展到150家，发明专利申请量增长10%，武汉理工大随州工业研究院投入运营。8家企业获省科技奖励，高新技术产业增加值突破110亿元，增长13%。以市校合作为载体，推动185家企事业单位与高校开展合作。全市吸引大学生实习实训5978人、就业创业3548人。市“人才超市”投入使用。

　　（四）投资底盘支撑强劲。围绕国家、省政策走向和投资方向，策划储备项目677个，投资额超省定目标2.2倍。深入开展项目建设季度拉练、集中开工、观摩晾晒等活动，全年新开工5000万元以上项目378个，亿元以上项目投资增长13%。汉十高铁随州段全线铺轨，投资180亿元的高铁新区拉开框架，随州加快迈进高铁时代。鄂北水资源配置一期工程基本完工，220千伏江头店输变电、烈山变电站扩建工程顺利投运。犇星化工扩能、东合汽车产业园、毅兴智能滤波器等重大项目建成投产，常森汽车部件、红色江山导航陀螺仪、湖北香思里仓储加工物流、随州农副产品物流园三期等项目进展顺利。投资结构不断优化，工业投资持续向好，增长14.9%，汽车机械投资增长24.5%。民间投资增长16.1%。招商引资取得成果，新签约项目188个，引进亿元以上项目49个。

　　　　（五）深化改革增添活力。供给侧结构性改革释放红利，为企业减税降费退税21.9亿元。深化“互联网+放管服”改革，“一网通办”实现省市县三级联通，建成“神农云”信息平台。推动完善金融市场体系和投融资体制改革，金融机构贷款余额增速全省第三，贷存比提高2.08个百分点，随州连续9年获评省金融信用市州。128个村（社区）完成集体产权制度改革，30多万村（居）民成为股东。全市6家国有林场改革通过省级验收。机构改革平稳推进，国税地税顺利合并，退役军人事务局、随州海关相继成立。

　　（六）城乡面貌继续改善。加快推进国家新型城镇化综合试点建设，城镇化率达到52.12%。城市功能不断完善。坚持规划引领、市区一体，大力实施主城区重点项目， 水一桥、涢水一桥、绕城南路建成通车，市委党校新校区等项目基本建成，武汉城市圈环线高速大随至汉十段、浪河至何店一级公路路基全线贯通，桃园大桥、汉东东路、南外环一级公路等项目加快推进。实施改善城区交通环境四年行动计划，畅通聚玉街等城市微循环，新增停车位2200个，建成2个立体停车场。城东生态补水、城北污水处理厂及管网建设、市污水处理厂提标、烈山大道中段改造相继完成，桃园公园、花溪河湿地公园一期等城市公园游园建成开放，新增绿地60万平方米，完成花溪小渠、南郊2号和3号渠黑臭水体治理。发挥数字城管作用，开展市容环境综合整治，拆除违建4万平方米。随县烈山湖大桥建成通车，广水市印台山文化生态园建成开园。美丽乡村加快建设。启动编制乡村振兴战略规划，淅河镇、洪山镇、长岭镇连续两年入围全国综合实力千强镇，何店镇、郝店镇获评湖北森林城镇，新增1个全国改善农村人居环境示范村、1个中国传统村落和5个省级美丽乡村示范村、82个绿色示范乡村。实施“四个三重大生态工程”，新改建各类厕所5.3万个，37个乡镇生活污水处理厂、31个乡镇垃圾压缩中转站基本建成，城乡生活垃圾无害化处理运行管理体系初步建立。精准灭荒3.8万亩，综合排名居全省前列。河湖长制全面落实。封江口国家湿地公园通过评估验收。

　　（七）民生福祉持续增进。坚持民生领域财政投入只增不减，支出占比81%，高于全省约6个百分点。就业形势总体稳定。城镇新增就业2万人，增发创业小额担保贷款9000万元，扶持创业724人，创业带动就业2691人，新增返乡创业主体2542个。社会保障力度加大。城乡医保政策整合实现“七统一”，参保人数达209.8万人，养老保险基本实现全覆盖，国家“医养结合”试点取得进展。企业退休职工养老金和城乡低保、特困人员供养稳步提标。保障性安居工程建设目标任务超额完成，城镇棚户区改造7236套、农村危房改造5897户。住房公积金制度保障作用充分发挥，综合服务平台建设在全省率先通过住建部验收。教育事业全面发展。投入1.3亿元改善义务教育办学条件，发放6916万元资助金。重视缓解主城区“大班额”问题，高新区云龙学校投入使用，鹿鹤小学、编钟学校启动建设。5名学子被北大、清华录取。文化体育活力彰显。早期曾国文物展、编钟音乐会等编钟出土40周年纪念活动圆满举办，大型刑侦电视剧《界线》在我市开拍。完善公共文化服务体系，全民阅读氛围日渐浓厚，新建村级文体广场100个，广水市获评省民间文化艺术之乡。全面实现“村村响”“户户通”，完成中央、省、市电视节目无线覆盖任务，市民可免费收看近20套节目。66项群众体育赛事首次实现政府购买服务。健康随州加快建设。城市公立医院改革、分级诊疗、医联体建设加快推进，市中心医院文帝院区投入使用，市中心血站新站完成主体工程。社会治理水平提升。村（社区）“两委”换届圆满完成，曾都区获评全国法治县(市、区)创建活动先进单位。平安随州建设扎实推进，“两抢一盗”案件大幅下降，安全生产、食药监管、信访维稳等工作得到加强。“双拥”创建积极推进，完成7.6万退役军人和其他优抚对象信息采集工作。精神文明建设取得成效。3人获评“中国好人”，4人荣登“荆楚楷模”，“光满·爱心红丝带”党员服务队获评全国学雷锋活动示范点。

　　（八）自身建设得到加强。推进政府系统党的建设，切实履行全面从严治党主体责任和党风廉政建设“一岗双责”。认真贯彻中央、省委省政府决策部署和市委工作要求，落实习近平总书记视察湖北重要讲话精神和省委《决定》重点工作清单。推进法治政府建设，修订《市政府工作规则》，不断规范行政行为。办理人大建议122件、政协提案114件，有效解决了一批群众关心、社会关切的热点难点问题。狠抓作风建设和政务服务质效提升，通过“双评双治双促”、作风聚焦栏目、电视问政等多种形式，纠治形式主义、官僚主义顽疾，查处突出问题276个，处理336人。

　　国家安全、国防动员、民防、消防、民族宗教、外事侨务、对台、援疆工作扎实推进，工会、共青团、妇女儿童、残疾人、老龄和慈善事业不断加强，审计、档案、保密、气象、烟草、机关事务、无线电管理、公共检验检测等工作取得新的成绩！

　　各位代表！看似寻常最奇崛，成如容易却艰辛。一年来的发展成绩来之不易，这是习近平新时代中国特色社会主义思想科学指引的结果，是省委省政府和市委坚强领导的结果，是市人大、市政协监督支持的结果，是全市广大干部群众团结奋斗、实干苦干的结果！在此，我代表市人民政府，向全市人民，向各位人大代表、政协委员，向各民主党派、工商联、人民团体和无党派人士，向驻随部队、武警官兵，向所有关心、支持、参与随州改革发展的各界朋友，致以诚挚的敬意和衷心的感谢！

　　我们也清醒看到，我市改革发展和政府工作面临的困难挑战还很多。主要是：发展不平衡不充分的问题较为突出，区域、行业、企业分化的趋势仍在延续，外贸出口等部分指标增速放缓；新旧动能接续不畅，创新能力不足，传统产业转型不够，新兴产业势强力弱，还不能很好适应高质量发展的要求；民生领域存在短板，教育、医疗、养老、环保等公共服务还不能很好满足群众日益增长的美好生活需要；一些干部担当不够、干劲不足，“四风”新表现一定程度存在，政务环境有待进一步优化。对此，我们一定高度重视，切实加以解决。

二、2019年重点工作

　　2019年是新中国成立70周年，是全面建成小康社会的关键之年。当前，我国经济发展健康稳定的基本面没有改变，支撑高质量发展的生产要素条件没有改变，长期稳中向好的总体势头没有改变，仍处于重要战略机遇期。特别是中央经济工作会议召开后，一系列政策举措合力齐发，已形成新的政策窗口期。近年来，省委省政府开创性谋划一系列重大战略，在我市形成叠加效应，“圣地车都”、特色产业增长极、鄂北生态屏障建设取得成效，一批重大项目落地实施，创新转型步伐加快，积极因素正在累积，内生动力逐步激发。我们要清晰把握时代发展的大方向，准确认识经济发展的大逻辑，既正视“成长的烦恼”，更保持发展的定力，抢抓机遇，乘势而上，奋力谱写新时代高质量发展新篇章！

　　今年政府工作的总体思路是：以习近平新时代中国特色社会主义思想为指引，深入贯彻党的十九大和十九届二中、三中全会以及习近平总书记视察湖北重要讲话精神，认真落实省委十一届四次全会及全省经济工作会议、省“两会”、市委四届五次全会及全市经济工作会议部署，坚持稳中求进工作总基调，坚持新发展理念，坚持高质量发展根本要求，坚持以供给侧结构性改革为主线，坚持深化市场化改革、扩大高水平开放，全面推进“一芯驱动、两带支撑、三区协同”战略实施，继续打好三大攻坚战，激发微观主体活力，着力稳就业、稳金融、稳外贸、资、稳投资、稳预期，统筹做好稳增长、促改革、调结构、惠民生、防风险各项工作，推进农业现代化、工业智能化、服务高端化、市域城镇化，保持经济持续健康发展和社会大局稳定，全力打造产业新城、文旅名城、生态绿城，建设品质随州，为建设特色产业增长极、构筑鄂北生态屏障和全面建成小康社会收官打下坚实基础。

　　今年经济社会发展主要预期目标是：地区生产总值增长7.8%—8%，规模工业增加值增长8%左右，固定资产投资增长11%左右，社会消费品零售总额增长11%左右，地方财政公共预算收入增长8%左右，外贸出口增长8%左右，实际利用内联资金、利用外资分别增长7.8%左右和8%左右，居民收入增长与经济增长基本同步，物价形势保持稳定，全面完成省下达的节能减排任务，生态环境进一步改善。

　　重点抓好六个方面工作：

　　（一）突出定向精准施策，奋力打好三大攻坚战。把准主攻方向，强化问题导向，抓住主要矛盾，持续打好攻坚之战，确保高质高效完成年度目标任务。

　　坚持防化并重，防范化解重大风险。健全政府债务、金融、社会风险预警化解机制。强化政府债务预算管理、绩效管理，有效遏制、化解隐形债务。坚决打击非法集资、网络借贷和非法金融活动，支持银行有序化解不良贷款。咬定三年目标，完善策略方法，保持扫黑除恶强大攻势。继续严打盗抢骗、黄赌毒等违法犯罪，全面防范化解公共安全、网络安全、社会稳定等领域的风险。

　　坚持准实并重，推进精准脱贫。聚焦深度贫困地区和特殊贫困群体，动态管理，扩大成果，确保完成5万人脱贫、50个贫困村出列。健全稳定脱贫长效机制，减少和防止贫困人口返贫。拓宽特色产业扶贫路径，以市场为导向，合理布局脱贫产业和项目。深入推进就业、教育、健康和社会保障兜底扶贫，全面完成贫困户危房改造、安全饮水工程存量任务。加大扶贫资金整合力度，落实扶贫小额贷款政策，推动贫困村基础设施建设。大力实施贫困村提升工程，深入推进“百企帮百村”活动，引导社会力量参与扶贫。推广用好中国社会扶贫网、随州扶贫云平台。扎实开展领导干部遍访贫困对象活动，加大驻村帮扶力度，强化扶贫领域作风整治，加强考核评估，倒逼脱贫攻坚责任落实。

　　坚持严管并重，加强污染防治。按照源头严防、过程严控、后果严惩的思路，继续推进九大攻坚行动，强化环保突出问题整改，进一步优化“气质”、提升水质、改善土质。抓好大气污染防治，综合治理扬尘、汽车尾气、工业废气、秸秆燃烧等，确保空气质量优良率达到80%以上。抓好水体污染防治，开展入河排污口、沿河化工企业、畜禽养殖场等专项整治，完成市县两级生活污水处理厂提标升级，确保国考断面水质全面达标。加强城市水环境综合整治，加快东西护城河、合溪渠改造，新增治理主城区2条黑臭水体。抓好土壤污染防治，强化农业面源、重金属污染治理，严厉打击非法处置危险废弃物行为。

　　（二）突出优化供给结构，奋力打造产业新城。全面贯彻“巩固、增强、提升、畅通”八字方针，深化供给侧结构性改革，提升供应链、产业链、价值链水平，加快建设特色产业增长极，促进产业有品质的成长。

　　大力推动制造业高质量发展。围绕助推汉孝随襄十制造业高质量发展带建设，制定重点产业高质量发展计划。加快发展新能源、轻量化、智能化专汽产品，推动专用车底盘、关键零部件配套协作发展，力争汽车机械产业产值增长10%以上，支持齐星集团打造百亿企业，提升中国专汽之都竞争力。加快国家应急产业示范基地建设，实施应急产业发展规划，抓好创新驱动转型、产品丰富拓展、质量品牌培育、开放重组优化、配套体系构建、应急救援服务等六大工程，重点推进高端消防、高空作业、电力抢险、防洪排涝、融冰清障及军工等专汽项目建设，力争应急产业产值突破200亿元。推动农产品加工业扩大规模，加快开发即食型、功能型、保健型产品，延长产业链，提高附加值，力争产值增长7%以上。引导风机产业差异化发展，提高市场占有率。实施“百企百亿”技改工程，持续支持企业“机器换人”，推进增品种、提品质、创品牌。对接全省“万企上云”工程，加快部门应用系统迁移“神农云”信息平台，拓展物联网、大数据、云计算等现代信息技术在传统产业的集成运用，促进制造业改造升级。

　　大力培育新兴产业。对接长江绿色经济和创新驱动发展带建设，重点推动新能源、晶体材料、高端装备、生物医药等产业发展，积极实施风力、生物质发电等项目，加快发展新能源装备制造，推进燃气储存设施建设。发挥泰晶科技等龙头企业引领作用，提高电子元器件研发生产能力。支持发展高端数控机床、工业机器人、智能输送设备等装备，促进新一代人工智能广泛应用。有序扩大中药材种植面积，加快开发中医药系列产品。

　　大力提升现代农业。深入实施乡村振兴战略，深化农业供给侧结构性改革，优化农业产业体系、生产体系、经营体系。加快“1+3+N”产业布局，壮大香菇优势产业，集中制袋达到3000万袋，规模化种植达到5000万袋，推进精深加工，完善菌种繁育、技术推广、质量监管体系，把“随州香菇”建成全省领先、全国一流的特色农产品优势区；夯实畜禽、优质粮油、茶果菜3个基础产业，巩固提升粮食产能，力争优质稻面积突破130万亩；加快发展洪山鸡、吉阳大蒜、泡泡青、马铃薯、优质桃、小龙虾等“N”个地方特色产品，新增有机农产品、绿色食品和农产品地理标志产品10件以上。推广虾稻共作、稻渔种养等绿色高效发展模式。持续壮大油茶、兰草、蓝莓、花卉苗木等基地规模，新增特色经济林和林下经济面积3万亩。引导土地经营权流转，因地制宜发展适度规模经营，新培育新型农业经营主体300家。深化一二三产业融合，扶持培育休闲农业、森林康养、农村电商等新产业新业态。继续推进“三乡工程”，促进城乡资源要素有序流动，抓好新型职业农民培育工程，为现代农业注入新血液新动能。

　　大力发展民营经济。坚定不移贯彻“两个毫不动摇”，全面落实降本减负等支持民营企业发展的政策措施，面对面、心贴心、实打实服务企业，精准有效支持民营经济做强做优。抓好中小企业成长工程和企业家培育计划，制定“3个30”帮扶方案，重组盘活30家困难企业、跟踪服务30个过亿元重大增长点、新增30家规模企业。落实纾困专项基金，增加市金控集团担保基金，从信贷、发债、股权融资等方面发力，缓解民营企业融资难题。进一步降低企业税费、社保、用能、物流及制度性交易成本，强化人才、信息、土地、资本、技术等要素供给。建立企业家参与涉企政策制定机制。

　　大力支持县域经济。坚持全要素、全产业链、全地域谋划，积极对接融入全省“一芯驱动、两带支撑、三区协同”战略布局，打造县域经济升级版。支持随县做优特色产业发展、能人回乡兴乡、文化传承创新、美丽乡村建设、基层党建引领五篇文章，推动乡村振兴。支持广水市推进产业、空间、城乡、军民、区域五个高品质融合，实现高质量发展。支持曾都区提升产业品质、建管品质、环境品质、生活品质，建设品质随州示范区。支持随州高新区当好创新驱动发展、绿色低碳发展、产业集聚发展、服务促推发展、拼搏赶超发展“五个表率”，助力全市产业新城建设。支持大洪山聚力建设高品质景区，助力全市文旅名城建设。推动各级各类开发区和工业园区产城融合，提升功能配套水平。

　　大力增强创新能力。引导企业聚焦主业、练好内功，加强技术创新、品牌创新、产业创新、市场创新。支持省专汽研究院、香菇研究院和武汉理工大随州工业研究院等平台开展科技攻关，推动成果转化。优化创新环境，完善激励政策，保护企业知识产权。深入推进各领域创新创造，健全支撑平台和服务机制。加强高新技术企业培育，力争高新技术产业增加值增长10%以上。实施随州工匠、名医名师名导等工程，加大柔性引才力度，培育引进一批高层次高素质人才。强化人才服务，加快各类人才创新平台建设，提升市“人才超市”运营服务水平。持续深化市校合作，深入开展“千企联百校”和高校院所专家行活动，积极促成更多高校来我市建立分支机构、研发中心，推动“一园十基地”建设取得更大成效。

　　大力促进消费提质扩容。实施服务业提速升级行动计划，加快发展文体娱乐、健康养老、家政服务、教育培训等生活性服务业，突破性发展研发外包、工业设计、人力资源等生产性服务业，满足多样化消费需求。鼓励发展商贸流通和电子商务，打造线上线下协同互动的消费生态。积极引进商业综合体，加快明珠新城商业综合体建设，推动万达广场开门营业。推广发展连锁经营、统一配送模式，抓好物流园区建设，构筑现代物流体系。

　　大力夯实投资底盘。坚定不移把项目投资作为经济工作的主抓手、主战场，持续三年开展“重大项目建设年”活动，推行每旬一通报、每月一调度、每季一观摩、半年一考核、年终结硬账和定期集中开工的项目建设工作机制，实行重点项目定时、定向、定项调度，全力扩大有效投资。超前谋划事关长远发展的重大布局、重大产业、重大项目，抓好储备库、建设库、达效库“三库”建设，积极对上争取无偿补助和专项债券支持，确保储备库项目投资额达到上年固定资产投资的2.5倍，转化率达到30%以上。强力协调推进重大项目，做实随州至信阳高速公路前期工作，抓紧鄂北水资源配置配套工程、电力主网及农村电网升级改造、南北外环一级公路等项目建设。加快黄鹤楼酒业搬迁、马可迅铝轮、品源食品加工等重点产业项目实施，推动常森汽车部件、程力二期等项目投产见效，确保工业投资占比40%以上。进一步放宽领域，激发民间投资活力。

　　（三）突出文化创新发展，奋力打造文旅名城。注重盘活特色资源，讲好随州故事，推动文明传承、文化延续和文旅融合，致力建设“武汉后花园”、中国优秀旅游目的地、世界华人谒祖圣地。

　　擦亮城市名片。增强文化自信，实施文化品牌战略，推动炎帝文化、编钟文化、曾随文化、红色文化等地域文化创造性转化、创新性发展。加强国家历史文化名城建设，编制曾（随）文化大遗址走廊保护利用规划和擂鼓墩国家考古遗址公园规划、历史文化街区保护规划，加强重点文保单位和文化遗址的保护管理，加快擂鼓墩驻军部队搬迁，启动市博物馆“汉东大国”陈列布展，谋划建设青铜小镇，推进编钟音乐文化之都建设，争创湖北中华优秀传统文化传承发展工程示范区。深度开发特色文创产品，促进传统工艺提高品质。扶持发展各类文化市场主体，培育新型文化业态，推动文化产业高质量发展。

　　发展全域旅游。强化“旅游+”“+旅游”资源整合联动，大力发展全域旅游、全业旅游，培育生态文化旅游战略性支柱产业。重点打造随南生态文化养生旅游区、随西民俗休闲旅游区、随北山地探险养生旅游区、随中产业融合示范区、随东“关寨”生态山水观光区五大片区。办好己亥年寻根节。加强炎帝故里文化旅游区、田王寨、桃源村、印台山文化生态园等高等级景区创建，支持随县创建全国全域旅游示范区、大洪山打造国家级旅游度假区。大力发展观光农业、特色民宿等乡村旅游，打造一批田园综合体。加强宣传推介，探索开展“拼盘营销”，提升炎帝故里、编钟之乡、全国十佳魅力城市等品牌知名度、美誉度，力争全年接待游客2800万人次、旅游综合收入178亿元。

　　推进文明创建。大力培育践行社会主义核心价值观，深入开展群众性精神文明创建活动，倡导移风易俗，全面提高市民文明素质和社会文明程度，积极争创全国文明城市。健全公共文化服务体系，加强文化馆、图书馆等建设，新建100个村级文体广场，开展全民阅读、送戏下乡、戏曲进校园等活动，不断丰富群众精神文化生活。广泛开展群众性文化体育活动，推进体育场馆建设，建立一批机关事业单位、学校体育场地周末及节假日免费开放试点，做好第七届世界军人运动会火炬传递工作。

　　（四）突出构筑鄂北屏障，奋力打造生态绿城。坚持不懈走生态优先、绿色发展新路，推动绿色发展示范区建设，让绿色成为高质量发展和品质随州的鲜明底色。

　　实施生态工程，全面加强修复保护。坚决管住斧头、守住山头、护好源头，统筹山水林田湖草系统治理，深入推进“四个三重大生态工程”，争创省级生态文明建设示范市。深化“厕所革命”，新改建各类厕所4.5万个。强化生态修复，灭荒造林3.5万亩，完成矿山复绿890亩，严防重大林业有害生物疫情蔓延。加快徐家河、淮河国家湿地公园试点建设，完成省第五次森林资源普查，做好国家森林城市复查迎检工作。提升河库水质，推动河湖长制从“有名”到“有实”转变，深化府澴河流域水生态综合治理，重拳打击非法采砂行为，依法加强城乡饮用水源地保护，确保37个乡镇污水处理设施平稳运行。强化垃圾治理，坚持减量化、资源化、无害化，推行生活垃圾分类，探索建设配套体系。推进餐厨垃圾集中处理设施建设，尽快实现城乡生活垃圾无害化处理全达标，建成城区垃圾无害化处理设施。

　　补齐补强短板，全面建设美丽乡村。遵循乡村发展规律，抓紧编制村庄规划，加强古村落古民居保护与利用。加快补齐农村基础设施和公共服务短板，持续改善供水、供电、交通、通讯等生产生活条件。推进水利工程建设，建成高标准农田11.1万亩，加快“四好农村路”建设，新改建农村公路350公里。实施农村人居环境整治三年行动，提升田水路林村风貌。推进大洪山长岗、应办多肉艺术等特色小镇建设，争创一批省级绿色示范乡村。

　　推动低碳发展，全面倡导绿色生活。严格项目准入，加强工业节能调控监察。开展低碳园区、清洁生产试点示范企业创建，推进公共环保型涂装车间建设，支持企业发展循环经济，加快化工企业搬迁入园。继续抓好“退二进三”。完成第三次全国土地调查，积极争创国土资源节约集约示范市。积极倡导绿色生活方式，引导全社会形成崇尚节俭、低碳绿色的消费理念。

　　（五）突出深化重点改革，奋力打造开放高地。坚持问题导向和目标导向相统一、试点先行和全面推进相促进，把新时代改革开放推向深入。

　　深层次推进改革。以经济体制改革为重点，推动相关改革走深走实，激发微观主体活力和创造力。高质量完成政府机构改革任务，优化政府机构设置和职能配置。深化“放管服”改革，加快数字政府建设，推进“互联网+政务服务”和“一网一门一次”改革，实现省市县乡村五级联通，让网上办成为常态，全面优化营商环境。抓好“证照分离”改革，全面推广“多评合一、多审合一、多验合一、多证合一”，优化审批流程。积极发展多层次资本市场，深化投融资体制改革，推进政府投融资平台公司专业化、市场化转型，加强政银企对接，支持企业上市，提高金融体系服务实体经济的能力。对接央企、省企，促进与本地企业合作重组，加快发展混合所有制经济。推进农村集体产权制度改革，建好曾都区国家级试点，完善“三权分置”制度，推动农村资源变资产、资金变股金、农民变股东。深化综合执法、国资国企、财税体制、价格机制等改革。

　　多途径扩大外贸。抢搭国家全方位开放快车，积极参与“一带一路”建设，推动出口市场多元化，不断扩大朋友圈、贸易圈。靶向扶持外向型企业，引导国内外市场双向突破，既支持开展一般贸易，又鼓励扩大服务贸易出口，降低市场风险。深化国际产能和制造合作，优化出口商品结构，建好全国最大的食用菌出口基地和省级茶叶、中药材、生物医药、汽车及零部件出口基地。推动随州海关完善口岸功能，加快建设保税物流中心（B型），促进贸易便利化。

　　大力度招商引资。坚持把招商引资作为“一号工程”，大员上阵、全员参与，扎实开展招商、安商、暖商、利商行动。围绕特色产业补链强链，聚焦高新、精准择优引进上下游关联企业，力争每个特色产业引进3个以上亿元项目。积极策划重大招商活动，办好寻根节经贸洽谈会。深化区域合作招商，建好武汉经开区随州产业园。创新督办、考核、落地等招商工作机制，确保引进过亿元项目45个以上。

　　（六）突出增进民生福祉，奋力创造美好生活。坚持以保障改善民生为出发点和落脚点，加快建设有品质的城市，提供有品质的公共服务，不断提升全市人民的幸福指数。

　　进一步加强城镇建管。坚持精心规划、精致建设、精细管理，下足“绣花”功夫，着力提升城市功能品质和核心竞争力。坚持一张蓝图绘到底，统筹推进“多规合一”。多方筹措资金，建设主城区重点项目80项。完善高铁新区基础工程，推进汉十高铁随州南站、随县站建设，启动高铁站前商务区建设，建成站前广场及广庆路、广贺路、站前北路等项目，保障汉十高铁顺利通车，把高铁新区打造成产城融合的样板和新的经济增长极。实施公共设施工程，启动新建城南新区片区路网二期、亚鑫路等道路，完成桃园大桥、明珠一路等项目建设。加快老火车站和草店子城市综合体项目实施，建成市委党校新校园、随州农副产品物流园三期等项目。推进“城市双修”，加快地下综合管廊和海绵城市建设，改造老城区易涝点和人行道，推进背街小巷硬化、绿化、亮化。加快涢水、漂水河等公园游园建设，织密、延伸城市绿道，新增城市绿地45万平方米。深化综合治理工程，坚持市区一体、重心下移，推进城管体制和环卫管理体制改革，创建全国卫生城市。开展占道经营大清理、市政设施大整修、门前三包大提升、不文明行为大劝导等清洁家园“八大行动”，扫干净、守秩序、保畅通、树形象。突出抓好停车服务和管理，完善交通信号控制系统，加强停车场等配套设施建设，增加一批临时停车位，推动智能停车系统建设。优化调整公交线路，严查超载超限，推进出租车行业自律。加强小区物业规范化管理，继续开展物业服务标准化试点建设。持续改善老旧社区基础设施。健全责任体系，加大违建打击力度。大力支持随县新县城、广水马都司生态新城完善功能和提升品质，支持集镇基础设施提档升级。

　　进一步促进就业创业。把稳就业摆在突出位置，实施就业优先战略。落实更加积极的就业政策，重点解决高校毕业生、农民工、退役军人、就业困难人员等群体就业。推进就业提升和技能展翅计划，加大稳岗支持力度，确保全市新增就业1.6万人。抓好返乡创业三年行动计划，加强创业孵化平台建设，用好创业担保贷款，激发大众创业活力，实现返乡创业2500人。

　　进一步强化社会保障。精准推进全民参保计划，重点向中小微企业、农民工和灵活就业人员扩面。抓好国家“医养结合”试点建设，巩固“两室联建”成果，建成一批农村幸福院试点，不断完善养老护理体系。强化福利机构管理，提高特困人员、孤儿集中供养质量，建好市流浪乞讨人员救助管理站。加强农民工工资支付保障。深入实施安居工程，全市改造棚户区3016户，公租房分配入住率达到90%以上，其中主城区改造棚户区1975户。完成住房公积金扩面6000人，新增归集8亿元以上，支持新市民住房消费。完善住房市场体系，推进房地产健康发展。

　　进一步发展各项事业。坚持办好人民满意的教育，加快推进教育现代化。推进学前教育三年行动计划，多渠道增加普惠性学前教育资源供给。促进义务教育优质均衡发展，继续消除“大班额”，迁建五丰学校。改善普通高中办学条件，深入实施高中阶段教育普及攻坚计划。加快发展职业教育、特殊教育，规范发展民办教育。筹措6000万元，资助6万人次家庭困难学生。推进健康随州建设，深化医药卫生体制改革，坚持中西医并重，加大医药、医保、医疗“三医”联动和公立医院、基层卫生综合改革力度，健全现代医院管理制度，加强医联体建设。推进市妇幼保健院新院区建设。促进计划生育转型发展，完善和落实全面二孩配套政策，建成60个标准化公共母婴设施。

　　进一步创新社会治理。坚持和发展新时代“枫桥经验”，推动建立自治、法治、德治相结合的现代基层社会治理体系。加强立体化、信息化社会治安防控体系建设，新增一批主城区A类公共视频探头。优化社区网格化管理，依法有序组织居民参与社区治理和小区管理。完善乡村治理体系，培育良好家风、文明乡风、淳朴民风。健全公共法律服务体系，推进普遍建立法律顾问制度，拓展法律援助覆盖面。支持工会、共青团、妇联等群团组织更好发挥作用，大力发展社会组织，开展志愿服务。加强应急管理体系建设，提升防灾减灾救灾能力。树牢安全发展理念，落实企业主体责任和政府监管责任，筑牢安全防线，坚决遏制重特大安全事故发生。加强食品药品监管，让人民群众饮食用药安全放心。做好第四次全国经济普查工作。加快信用体系建设，全力打造公平公正的信用随州。深入开展国防动员，扎实做好退役军人事务工作，推动军民融合深度发展。

三、加强政府自身建设

　　新时代是干出来的。面对新的赶考路，必须不断加强政府自身建设，干字当头，拼搏赶超，努力建设人民满意的服务型政府。

　　（一）忠诚执着干事。坚持党的全面领导，把对党忠诚作为第一位的政治要求，用习近平新时代中国特色社会主义思想武装头脑，树牢“四个意识”，坚定“四个自信”，做到“两个维护”，坚决落实省委省政府和市委各项决策部署。严格规范党内政治生活，深入开展“不忘初心、牢记使命”主题教育，把稳思想之舵，补足精神之钙。坚守人民至上，全心全意办实事，尽心尽力解民忧。

　　（二）依法履职干事。坚持法治为上、循法而行，全面贯彻实施宪法，积极推进相关领域立法工作。把依法行政贯穿政府工作全过程，健全完善行政决策机制，严格依法用权、依规办事。规范行政执法自由裁量权，推进政务服务标准化。坚持强化监督、阳光行政，自觉接受人大法律监督、工作监督和政协的民主监督，主动接受社会各方面监督，广泛听取人民团体的意见，提高建议提案办理实效，更加注重在监督下工作、在监督中履职。

　　（三）担当提能干事。大兴勇于担当之风，树立重实干、尚实绩的鲜明导向，敢啃硬骨头、勇挑重担子，善始善终、善作善成抓落实。着力增强“八个本领”，努力从政治、历史、全局的高度思考问题，锻造专业能力、专业精神。大力倡导“智圆行简、实心实政”的工作作风，增强工作的科学性、预见性、主动性和创造性。严格督办考核，强化季度综合督查、述职评议、晾晒约谈，严防弄虚作假，加强问效追责。坚持宽严相济，强化正向激励，让改革者轻装上阵、担当者脱颖而出、实干者大显身手。

　（四）清廉干净干事。持之以恒强基固本，推动全面从严治党向纵深发展、向基层延伸，认真履行主体责任，不断优化政治生态。持之以恒正风肃纪，严格落实中央八项规定及其实施细则精神，深化“双评双治双促”活动，坚决破除形式主义、官僚主义。持之以恒反腐倡廉，大力整治群众身边的腐败问题。持之以恒勤俭理政，保持“三公”经费只减不增，保障民生支出只增不减，营造清廉干事的良好形象。

　　各位代表，奋进新时代，开启新征程！让我们紧密团结在以习近平同志为核心的党中央周围，在省委省政府和市委的坚强领导下，坚定信心，务实重行，凝心聚力，携手前进，为建设特色产业增长极、构筑鄂北生态屏障、建设品质随州不懈奋斗，以优异成绩迎接新中国成立70周年！
